# A TMS investigation on the role of the cerebellum in pitch and timbre discrimination

**DOI:** 10.1186/s40673-016-0044-4

**Published:** 2016-03-02

**Authors:** Carlotta Lega, Tomaso Vecchi, Egidio D’Angelo, Zaira Cattaneo

**Affiliations:** Department of Psychology, University of Milano-Bicocca, Milan, Italy; Department of Brain and Behavioural Sciences, University of Pavia, Pavia, Italy; Brain Connectivity Center, C. Mondino National Neurological Institute, Pavia, Italy

**Keywords:** Pitch, Timbre, Transcranial magnetic stimulation (TMS), Auditory discrimination

## Abstract

**Background:**

Growing neuroimaging and clinical evidence suggests that the cerebellum plays a critical role in perception. In the auditory domain, the cerebellum seems to be important in different aspects of music and sound processing. Here we investigated the possible causal role of the cerebellum in two auditory tasks, a pitch discrimination and a timbre discrimination task. Specifically, participants performed a pitch and a timbre discrimination task prior and after receiving offline low frequency transcranical magnetic stimulation (TMS) over their (right) cerebellum.

**Results:**

Suppressing activity in the right cerebellum by means of inhibitory 1 Hz TMS affected participants’ ability to discriminate pitch but not timbre.

**Conclusion:**

These findings point to a causal role of the cerebellum in at least certain aspects of sound processing and are important in a clinical perspective helping understanding the impact of cerebellar lesions on sensory functions.

## Background

The cerebellum is traditionally considered a “motor controller” and its role in the acquisition of motor skills is well established [1, 2]. Nevertheless, accumulating evidence suggests that the cerebellum may play a critical role in non-motor functions, contributing to cognitive and emotional processing [3–6]. In particular, a cerebellar involvement has been found in high-order cognitive processes such as language [7], working memory [8], and spatial processing [9, 10]. Furthermore, the cerebellum seems to play a role in the generation of sensory predictions [11, 12], optimising perception [13]. Accordingly, patients with cerebellar lesions are often impaired in processing visual information, such as in deciding speed and direction of moving stimuli [14]. Neuroimaging evidence also shows that the cerebellum is significantly active in neurologically unimpaired individuals during sensory discrimination, such as visual (and auditory) motion discrimination [15]. Also, interfering with cerebellar activity via brain stimulation has been found to impact on visual processing [16, 17].

The cerebellum is also involved in auditory processing, where it plays a critical role in timing [18–21]. Cerebellar activations have also been observed in healthy subjects during passive listening of both music [22, 23] and speech [24, 25]. Moreover, pitch discrimination and melody discrimination [26–28], as well as sound intensity and duration discrimination [29], activate cerebellar regions. For instance, Petacchi and colleagues [23] showed that cerebellar activity significantly increased during a pitch discrimination task compared to passive listening, with the cerebellum responding more when the difficulty of the discrimination to be performed increased. Importantly, cerebellar activity during auditory discrimination tasks has been consistently observed even in the absence of any motor or cognitive component [30]. Patients’ findings support evidence obtained in healthy individuals: cerebellar disorders are often associated to deficits in melody recognition [31], in discriminating small differences in sound duration [32] and in pitch discrimination [33].

Although there is considerable evidence that the cerebellum contributes to auditory perception [30], the precise role of the cerebellum in different aspects of sound processing is not completely clear. In particular, whilst neuroimaging [23, 30] and patients’ [33] studies converge in indicating a role of the cerebellum in pitch processing, whether the cerebellum also contributes to other sound features such as timbre (i.e., the property of a sound which allows a person to distinguish musical instrument when pitch, loudness and duration remain identical), is less clear. Indeed, whereas some neuroimaging studies reported significant cerebellar responses to sound timbre [34, 35], in other studies investigating timbre processing cerebellar activations were not considered [36].

The aim of this study was to analyse the role of the cerebellum in pitch and timbre processing using transcranial magnetic stimulation (TMS). While neuroimaging techniques provide correlational evidence regarding the activation in a specific brain region during an ongoing cognitive process, TMS allows establishing the causal role of specific cortical areas in a given task [37, 38]. Moreover, participants in TMS experiments act as their own controls overcoming some of the limitations intrinsic in patients’ studies, such as potential differences in pre-morbid ability, and variability depending on high heterogeneity of lesions’ sizes and gravity. Specifically, in this study we applied off line low frequency repetitive TMS to induce transient suppression of cerebellar activity [39, 40] before participants’ performance in a pitch and timbre discrimination tasks. If the cerebellum is causally involved in pitch and timbre processing, participants should perform worse following real than sham (faked) stimulation.

## Methods

### Participants

Fourteen participants (9 F; mean age = 21.93 ys; SD = 1.86) took part in the experiment. All participants were right-handed [41] and had less than 3 years of formal musical training, as revealed by a self-reported history of musical experience. Prior to the experiment, each participant filled in a questionnaire (translated from Rossi et al. [42]) to evaluate compatibility with TMS. None of the volunteers reported neurological problems, familiarity for seizures nor was taking any medication that could interfere with neuronal excitability. Written informed consent was obtained from all participants before the experiment. The protocol was approved by the local ethical committee. Participants’ treatment was conducted in accordance with the Declaration of Helsinki.

### Stimuli

Stimuli used in the pitch discrimination task consisted of 21 pure tones (i.e., tones with a sinusoidal waveform, where the wave consists of a single frequency) of 200 ms generated through the software Audacity (http://audacity.sourceforge.net/). All tones had a frequency comprised between 1000 and 1200 Hz, and were presented at a level of 75 dB SPL. Stimuli used in the Timbre discrimination task consisted of 21 complex tones of 200 msec duration. Sound files in the timbre task were created from digitized samples of real musical instruments, with all instruments belonging to the wind or string family. Sound files used in the timbre task were taken from the University of Iowa Musical Instrument Samples (Lawrence Fritts, http://theremin.music.uiowa.edu/MIS.html).

### Procedure

**Fig. 1:**
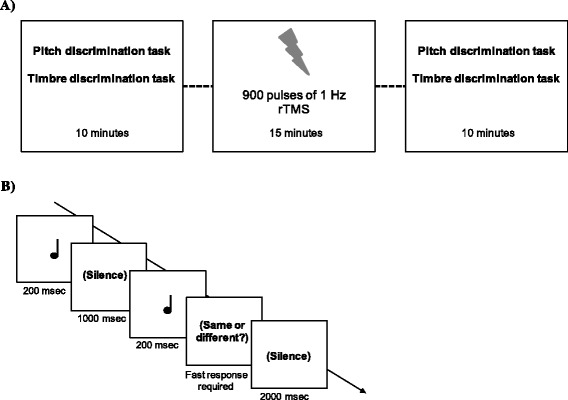
**a** The experimental paradigm: participants underwent two experimental sessions, one with real TMS, and the other with sham TMS (order of sessions counterbalanced). In each session, participants performed the task twice, once before and once after receiving 1 Hz repetitive 15 min TMS over the right cerebellum. **b** The timeline of an experimental trial. In the pitch discrimination task the two sounds were pure tones only differing for pitch. In the timbre discrimination task the two sounds were tones of the same instrumental family (wind vs. string) presented at the same high frequency and differing only in their timbre characteristic

Figure 1 shows the experimental paradigm (Fig. 1a) and the timeline of an experimental trial (Fig. 1b). Participants seated comfortably in a dimly lit room and stimuli were binaurally delivered through professional headphone (Sennheiser HD 280 Pro headphone). Each subject took part in two different sessions (Real and Sham) that were separated by an average of 6 days (range 5–7). In each session, participants performed both the pitch discrimination task and the timbre discrimination task twice: once before, and once after receiving 15 min of off-line 1Hz rTMS over the right cerebellum. During TMS, no task was performed and participants were instructed to minimise movements and be silent. The post-stimulation task started immediately after the end of the stimulation. Both the pitch and timbre discrimination tasks required participants to indicate by left/right key pressing using their dominant hand whether two consecutively presented sounds (separated by 1 sec of silence interval) were identical or different. Participants were instructed to respond as fast and as accurately as possible. Intertrial interval was 2 sec. In each task, 42 sounds were presented: in half of the trials the two sounds to be compared were identical, in the other half they were different. In the different trials of the pitch task, the second pure tone presented could be 20, 30 or 40 Hz higher (ascending trials) or lower (descending trials) compared to the first one. The number of descending and ascending trials was counterbalanced. In the timbre task, the two sounds to be compared in each trial were identical in terms of frequency and intensity, but they had different timbre. In particular, two different string sounds may be presented, or two different wind sounds (wind and string sounds were never presented in the same trial to avoid ceiling effects in recognition). Task order (pitch and timbre discrimination), TMS condition order (Real vs. Sham), and the response key assignment for same/different response were counterbalanced across participants. The software E-prime 2.0 (Psychology Software Tools, Pittsburgh, PA) was used for stimuli presentation, data collection and TMS triggering. Pre and post-stimulation task sessions lasted approximately 10 min (5 min for each task).

### Transcranial magnetic stimulation

TMS was administered over the right cerebellum by means of a Magstim Rapid^2^ machine (Magstim Co Ltd, Whitland, UK) with a 70 mm butterfly coil. An air-cooled coil was used, in order to avoid coil overheating. A fixed intensity of 45 % of the maximum stimulator output was used, in line with prior studies [43]. The right cerebellar hemisphere was targeted in light of previous evidence pointing to a right lateralized cerebellar activation during timbre processing [35], whereas pitch discrimination seems to induce bilateral cerebellar activations [23]. The right cerebellum was localised in each participant as the region located 1 cm under and 3 cm lateral to the inion as in prior studies [44, 45]. Prior studies using neuronavigated TMS have demonstrated that this point lies over the cerebellar hemisphere [17, 46]. The coil was placed over the right cerebellum with the handle pointing upward, parallel to the inion-nasion line [44, 47]. Previous studies have shown that rTMS at 1 Hz temporarily reduces the excitability of the stimulated cortex for a time window that outlasts the period of stimulation [39, 40]. Sham stimulation was conducted with the coil held at a 90° position in order to ensure that the magnetic field did not stimulate the target area. The stimulation paradigm in the sham condition was the same as that of real rTMS stimulation.

### Statistical analyses

Analyses were performed on mean accuracy scores and on mean reaction times (RT) for correct responses. Prior to analyses, reaction times 3 s.d.’s above or below the participants’ mean were removed (this corresponded to 1.33 % and 1.99 % of the trials in the pitch and timbre discrimination task, respectively). A repeated-measures analysis of variance (ANOVA) with TMS condition (real vs. sham) and Session (pre-stimulation vs. post-stimulation) as within-subjects factors was performed separately for the pitch and the timbre discrimination task on accuracy scores and correct RT. Bonferroni-Holmes correction was applied to post-hoc comparisons.

## Results

### Pitch discrimination task

**Fig. 2:**
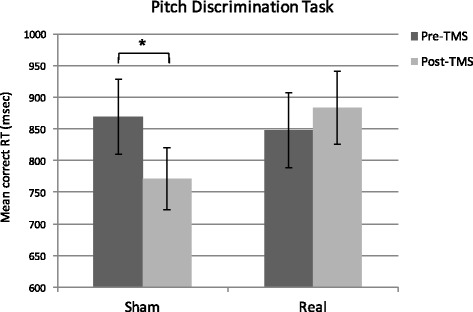
Mean response latencies for correct responses as a function of TMS (Real vs. Sham) and Session (Pre vs. Post stimulation) in the Pitch discrimination task. Participants were significantly faster (as indicated by the asterisk) in the post compared to the pre session when sham TMS was delivered, reflecting learning effects. RT were comparable in the pre and post real TMS sessions, suggesting that real TMS affected learning effects. Error bars represent ±1 SEM

Mean accuracy was above 73 % (SD = 9 %) in all the experimental conditions. Analysis on accuracy scores revealed no significant main effects of TMS, *F*(1,13) = 3.75, *p* = .08, η_p_^2^ = .22, and of Session, *F*(1,13) = 1.47, *p* = .25, η_p_^2^ = .10. The interaction TMS by Session was not significant, *F*(1,13) < 1, *p* = .92, η_p_^2^ = .00. Mean correct RT are shown in Fig. 2. The ANOVA on correct RT showed no significant main effect of either TMS, *F*(1,13) = 1.30, *p* = .27, η_p_^2^ = .09, or Session, *F*(1,13) = 2.86, *p* = .11, η_p_^2^ = .18. The interaction TMS by Session was significant, *F*(1,13) = 8.08, *p* = .01, η_p_^2^ = .38. Post-hoc *t*-tests revealed that participants were significantly faster in responding in the post-sham stimulation session compared to the pre-sham stimulation session, *t*(13) = 3.52, *p* = .016, reflecting learning effects. In turn, RT were comparable between pre-real and post-real stimulation sessions, *t*(13) = .63, *p* = .54, suggesting that real TMS interfered with learning. Moreover, whilst RT were comparable in the sham and real pre-sessions, *t*(13) = 1.24, *p* = .24, indicating a similar level of baseline performance, participants tended to be slower following real, *t*(13) = 2.31, *p* = .09 (*p* = .03 uncorrected), than sham TMS.

### Timbre discrimination task

**Fig. 3:**
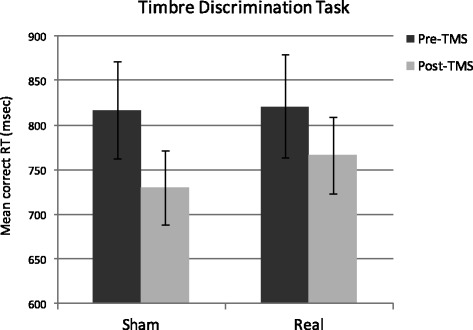
Mean response latencies for correct responses as a function of TMS (Real vs. Sham) and Session (Pre and Post stimulation) in the Timbre discrimination task. Participants were overall faster in the post compared to the pre session, reflecting learning effects. The type of stimulation (Real vs. Sham) did not affect performance. Error bars represent ±1 SEM

Mean accuracy was above 82 % (SD = 9 %) in all the experimental conditions. The ANOVA on accuracy scores revealed no significant main effect of either TMS, *F*(1,13) = .50, *p* = .49, η_p_^2^ = .04, or Session, *F*(1,13) = .16, *p* = .22, η_p_^2^ = .12. The interaction TMS by Session was not significant, *F*(1,13) < 1, *p* = .99, η_p_^2^ = .00. Figure 3 shows mean participants’ correct RT. The ANOVA revealed a significant main effect of Session, *F*(1,13) = 4.95, *p* = .04, η_p_^2^ = .28: participants were overall faster in the post-stimulation session (irrespective of stimulation being real or sham), reflecting learning effects. Neither the main effect of TMS, *F*(1,13) = .03, *p* = .87, η_p_^2^ = .00, nor the interaction TMS by Session, *F*(1,13) = .57, *p* = .46, η_p_^2^ = .04, reached significance.

## Discussion

In this study we aimed to shed light on the possible causal role of the cerebellum in auditory processing, and in particular in pitch and timbre discrimination, in light of prior neuroimaging and patients’ data suggesting a cerebellar involvement in processing of music and single sound features [31–33, 48]. We found that interfering with cerebellar excitability via offline low frequency TMS significantly affected pitch discrimination, whereas it had no effect on timbre discrimination. In particular, in the pitch discrimination task real TMS counteracted learning effects that emerged in the other experimental conditions as faster responses in the post compared to the pre stimulation sessions.

Our findings are consistent with prior neuroimaging and neuropsychological evidence suggesting that the cerebellum is involved in perceptual tasks [8, 49, 50], possibly monitoring the incoming sensory events to optimize perception [13, 51]. More specifically, our data add to previous studies that showed that discrimination of pitch and melody elicit activation of cerebellar regions [26, 28, 33], pointing to a causal role for the (right) cerebellum in processing pitch. Accordingly, resting state fMRI has shown functional connectivity between bilateral anterior cerebellum and the auditory cortex in the temporal lobes [52]. In line with this, the cerebellum and the lateral anterior temporal lobe appeared to be bidirectionally interconnected during a rhyming judgment task [53]. More in general, consistent evidence suggests that the cerebellum projects not only to motor, but also to somatosensory areas [6]. Still, it is important to consider that the type of stimulation we used may have only affected posterior cerebellar regions, given the deep position of the cerebellum [54]. Indeed, coil geometry seems to be an important factor in determining effective stimulation of deep cerebellar regions, with the figure of eight coil likely being suboptimal when targeting motor areas [54]. Nonetheless, cerebellar stimulation parameters similar to ours significantly affected perceptual [16] and cognitive (for instance, linguistic) functions in prior studies (e.g. [55, 56]), suggesting that the stimulation we used was able to interfere with neural activity in the cerebellar-cortical network subtending discrimination of sound features.

Although prior neuroimaging evidence also suggested a possible role for the cerebellum in timbre processing [34, 57], we did not find evidence for this in our study. On the one hand, the lack of TMS effect in the timbre discrimination task ensures that the effects we reported in the pitch discrimination task were not due to unspecific effects of TMS slowing down responses regardless of the specific task at play. On the other hand, it is possible that real TMS affects auditory discrimination task only when the task has a certain level of complexity. Performance accuracy was indeed overall higher in the timbre than in the pitch task. This is in line with prior literature showing that non-musicians are more sensitive in sound categorization to changes in timbre than to changes in pitch [58]. Interestingly, previous studies demonstrated a positive correlation between cerebellar activation and task difficulty [15, 23, 28, 59]. Moreover, the right cerebellum may be more important than the left in difficult auditory discrimination [57].

In interpreting our data, it is also worth mentioning that pitch and timbre processing may have a different degree of lateralization in the brain. There is evidence for a right hemisphere dominance in the temporal lobes for musical timbre discrimination [60, 61] related to a right hemispheric specialization in processing spectral sound features, that are critical for discriminating timbre differences [62–64]. In line with these findings, the left cerebellum may be more important than the right in timbre processing (cerebral cortex fibers mainly projecting to the contralateral cerebellar cortex [65]). However, other studies in infants [66] and in adults [35, 67] reported left hemispheric cerebral cortex engagement underlying perception of timbre change. Moreover, Reiterer and colleagues [35] showed a right cerebellar activation during timbre processing, speculating that pre-linguistic sound features (including timbre) may be represented by a complex network that connects Broca’s area and the right cerebellum. In turn, previous neuroimaging studies mainly indicate bilateral cerebellar activation during pitch processing [23, 26–28, 30, 34], although some degree of lateralization may occur depending on task complexity [29, 57, 68]. Evidences are thus not entirely consistent regarding lateralization of timbre and pitch processing. Future studies may address this issue by comparing the effect of left and right cerebellar stimulation on auditory discrimination.

## Conclusions

In sum, our findings show that the (right) cerebellum plays a causal role in pitch processing. Future research is needed to better clarify the role of cerebellum in other aspects of auditory processing, such as rhythm or complex melody recognition and discrimination. Moreover, level of expertise in determining the involvement of the cerebellum in auditory functions deserves consideration. In fact, prior studies showed greater engagement of the cerebellum in rhythm perception and synchronization in musicians compared to non-musicians [69, 70]. Musicians have been found to detect pitch changes and rhythmic irregularities faster and more accurately than non-musicians [71–73], an ability that may also depend on different cerebellar involvement. The relation between level of expertise and cerebellar involvement in perceptual functions is an important topic to which brain stimulation may significantly contribute. Finally, our results are important in a clinical perspective helping understanding the impact of cerebellar lesions on sensory and cognitive functions.
